# The Limitations of Reward Effects on Saccade Latencies: An Exploration of Task-Specificity and Strength

**DOI:** 10.3390/vision3020020

**Published:** 2019-05-11

**Authors:** Stephen Dunne, Amanda Ellison, Daniel T. Smith

**Affiliations:** 1 School of Psychology, University of Sunderland, Faculty of Health Sciences & Wellbeing, City Campus, Sunderland SR1 3SD, UK; 2 Department of Psychology, Durham University, Science Site, South Road, Durham DH1 3LE, UK

**Keywords:** saccade, conditioning, reward, learning, distractor, remote distractor, antisaccade, oculomotor, attention

## Abstract

Saccadic eye movements are simple, visually guided actions. Operant conditioning of specific saccade directions can reduce the latency of eye movements in the conditioned direction. However, it is not clear to what extent this learning transfers from the conditioned task to novel tasks. The purpose of this study was to investigate whether the effects of operant conditioning of prosaccades to specific spatial locations would transfer to more complex oculomotor behaviours, specifically, prosaccades made in the presence of a distractor (Experiment 1) and antisaccades (Experiment 2). In part 1 of each experiment, participants were rewarded for making a saccade to one hemifield. In both experiments, the reward produced a significant facilitation of saccadic latency for prosaccades directed to the rewarded hemifield. In part 2, rewards were withdrawn, and the participant made a prosaccade to targets that were accompanied by a contralateral distractor (Experiment 1) or an antisaccade (Experiment 2). There were no hemifield-specific effects of the reward on saccade latency on the remote distractor effect or antisaccades, although the reward was associated with an overall slowing of saccade latency in Experiment 1. These data indicate that operant conditioning of saccadic eye movements does not transfer to similar but untrained tasks. We conclude that rewarding specific spatial locations is unlikely to induce long-term, systemic changes to the human oculomotor system.

## 1. Introduction

An eye movement is a paradigmatic example of a visually controlled action, as the kinematics of a saccade are fundamentally driven by the visual properties of the stimulus to which the saccade is directed, such as its luminance, spatial frequency and contrast [1], and its spatial and temporal proximity to other stimuli [2,3]. However, the extent to which a stimulus’ visual properties guide eye movements can also be influenced by learning. For example, Thorndike’s law of effect holds that actions that produce a satisfying effect will be repeated [4]. This law is typically implemented by using rewards to shape behaviour such that desirable actions are emitted more frequently. In studies of oculomotor behaviours, reward-based learning has been successfully applied to shaping the kinematic properties of eye movements to reduce latency [5,6,7,8,9,10] and increase the accuracy [11] and peak velocity [12] of eye movements. However, although eye movements are fundamentally spatial in nature, the majority of the studies in human participants have examined the effect of rewarding non-spatial visual features such as shape or colour on the visual control of oculomotor actions [13,14,15,16]. These studies typically yield a negative correlation between saccadic latency and reward, such that saccade latencies are faster to stimuli associated with larger rewards, greater oculomotor capture by a distractor previously associated with a high expected value [10] and increased exogenous capture of the eyes based on the learned value of a reward [14,16]. Similar effects have been observed using more complex tasks such as the Remote Distractor task and the antisaccade paradigm. For example, Mccoy and Theeuwes (2016) have reported increased erroneous saccades towards distractors of increasing value, even though this was detrimental to reward pay-out in their study [17]. The behavioural effect of incentives on antisaccades has also been investigated in both adults [18] and adolescents [19]. These studies found that the presence of incentives increased the number of correct antisaccades, but at the cost of slower saccadic reaction times (SRTs). One study has analysed the effects of both positive and negative motivators on pro- and antisaccades [20]. Participants were presented with a motivational cue indicating a reward, penalty or no consequence (neutral), following which a circular target stimulus would appear. The use of motivational cues was found to reduce all saccadic latencies, but to a greater degree following reward cues relative to penalty cues. These studies are often interpreted as evidence that rewarding a specific stimulus enhances its salience [15,21], a view which is consistent with other research showing that rewards can have a profound impact on covert attention [21,22,23]. It is not entirely clear how persistent these changes are because very few studies measure the time-course of extinction. However, Chelazzi, Perlato, Santandrea, and Libera (2013) found that the effect of feature-based rewards on attention lasted several days [24], and Knight, Smith, Knight, and Ellison (2016) reported that explicitly instructing participants to value a specific stimulus feature (colour) led to an attentional bias that lasted at least 3 weeks [25], suggesting these effects can be very long-lasting. These studies have led to speculation that feature-based reward paradigms may have potential as therapeutic interventions for patients with neuropsychological disorders of attention, such as neglect [26].

In contrast to studies of human participants, in the non-human primate literature, there is a rich tradition of exploring the effects of rewarding features and spatial locations. For example, pairing a specific spatial location with a food reward yields shorter saccade latencies, which is linked to changes in activation in oculomotor centres such as the superior colliculus [9,27,28]. Manipulating the expected value [29] and size [30] of the reward also reduces saccade reaction times to the location associated with high value. This finding has been extended to humans with a number of papers focusing on varying the expected value of the stimulus at the start of trials but do not pair specific values with different features. Using a monetary reward that differed in magnitude, Milstein and Dorris (2007) rewarded participants for fast and accurate prosaccades to a single visual target [8]. Differing the value of the reward between target locations the authors observed faster saccade latencies to locations associated with larger rewards. Oculomotor capture was also found to be greater when distractors were presented at locations with a high expected value, suggesting the presence of saccade preparation towards high expected value locations prior to the onset of the movement goals. Similarly, we observed that rewarding one specific saccade direction led to faster and more accurate eye movements to that direction [31]. In an elegant study, Wolf, Heuer, Schubö, and Schütz, (2017) included trials with targets of varying reward value [32]. When participants were given a choice of two targets in opposite hemifields, one with a higher value than the other, the influence of reward increased with difficulty of choice. They concluded that the size of the effect of reward on saccades was dependent on participants having to make a choice between two potential saccade goals. However, unlike feature-based rewards, the effects of location-based rewards are relatively transient. For example, in one of the only studies to explicitly measure extinction effects, we observed that location-based facilitation of saccadic latency was extinguished after ~120 unrewarded trials [31]. Furthermore, the effect of rewarding spatial locations does not transfer from eye movements to exogenous covert spatial attention. This result was particularly surprising given the well-established links between covert exogenous attention and oculomotor control [33,34]. These studies indicated that the effects of rewarding spatial locations are more transient and more task specific than the effects of feature-based rewards. However, this conclusion may be premature, as no studies have specifically examined the transfer of location-based operant conditioning of eye movements to other eye movement tasks.

Recording participant eye movement data, the present series of experiments were designed to address this issue by examining the transfer between a reward training task involving simple saccades and more complex eye movement tasks. Utilising a previously described reward paradigm [31], where participants were rewarded for making a saccade to one of two potential target locations, Experiment 1 investigated the transfer of this effect to a Remote Distractor task. Three different trial types were employed: (1) a known distractor trial, where the distractor used was the same stimuli associated with reward feedback in the reward paradigm; (2) a novel distractor trial, where the distractor used was a novel stimulus; (3) a no distractor trial, where the target was presented on its own with no distractors. Experiment 2 uses the same reward paradigm to investigate the transfer of reward learning to an antisaccade task. This experiment addresses the extent to which changes in saccade metrics triggered by rewards generalised to both trained (prosaccades) and untrained (antisaccades) eye movements.

## 2. Materials and Methods

### 2.1. Participants

Experiment 1 contained twelve participants (8 female, 4 male; 19–25 years; mean age 20.8 years). 9 were right eye dominant. Experiment 2 contained twelve participants (11 female, 1 male; 20–31 years; mean age 23.7 years). 7 were right eye dominant. All participants were recruited from Durham University, had normal or corrected-to-normal vision and were naive regarding the purpose of the experiment. All participants gave their informed consent for inclusion prior to participation in the study. The study was conducted in accordance with the Declaration of Helsinki, and the protocol was approved by the Ethics Committee of Durham University (Ref: 14/25).

### 2.2. Apparatus

A Cambridge Research Systems ViSaGe graphics card was used to generate the stimuli. Stimuli were displayed on a 17-inch Eizo Flexscan Colour Display monitor with a refresh rate of 100 Hz. Responses were collected using a two-button button box. Participants’ eye movements were recorded using a Cambridge Research Systems eye tracker with a sampling rate of 160 Hz.

### 2.3. Stimuli

In the reward paradigm outlined in Experiments 1 and 2, participants were presented with a black (5 cm/2) 0.3° × 0.3° fixation cross in the centre of the screen on a grey background (23 cm/2). A white target stimulus 0.5° × 0.5° (20 cm/2) square was presented to the left or right of the fixation cross. The stimuli were presented 6.5° to the left and 3.7° upwards from fixation. On a rewarded trial, participants were presented with green text of ’10 p’ indicating reward feedback. This feedback had a luminance of 19.61 cm/2. On unrewarded trials, participants were presented with red text of ‘0 p’ which had a luminance of 19.69 cm/2.

In the Remote Distractor (RD) task in Experiment 1, participants were presented with a 0.7° × 0.7° fixation cross in the centre of the screen on a grey background. A target stimulus 1.0° × 1.0° circle was presented to the left or right of the fixation cross. A related distractor square and an unrelated distractor triangle were both 1.0° × 1.0° of visual angle. Target and distractor stimuli were presented 6.5° to the left or right and 3.7° upwards from fixation.

During the antisaccade task in Experiment 2, participants were presented with a black fixation cross (0.7° × 0.7°) in the centre of the screen on a grey (23 cdm^2^) background. A black target stimulus (0.8° × 0.7° outline rectangle) was presented to the left or right of the fixation cross. The target stimuli were presented 6.5° to the left or right and 3.7° upwards from fixation.

### 2.4. Procedure

#### 2.4.1. Experiment 1

**Figure 1:**
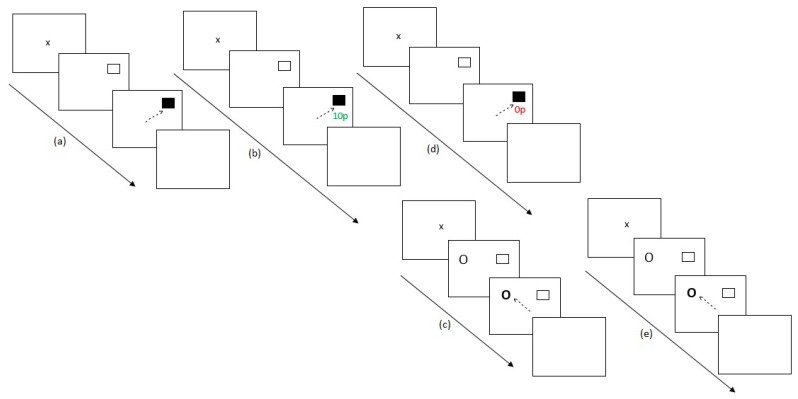
Experimental procedure for Experiment 1. (**a**) After assessing for eye dominance and a 9-point calibration procedure, participants completed 2 baseline blocks of the reward paradigm where no reward feedback was displayed. (**b**) Participants then completed 10 conditioning blocks of the reward paradigm where only one hemifield was rewarded at a 60% schedule. (**c**) Participants then switched tasks, completing six blocks of the remote distractor task to assess whether reward had any effect on this secondary unrewarded task. (**d**) A further six blocks of the reward paradigm were completed where no reward was given to participants for saccades. (**e**) Participants completed a final six blocks of the remote distractor task and the experiment ended.

Experiment 1 contained 30 blocks with participants switching between the two-eye movement tasks. Initially, participants completed the preconditioning phase (2 blocks) and the conditioning phase of the reward paradigm (10 blocks). Participants then completed the post-conditioning phase of the RD task (6 blocks), followed by the extinction phase of the reward paradigm (6 blocks) and finally the post-extinction phase of the RD task (6 blocks). Figure 1 displays this experimental procedure.

Prior to experimentation, eye dominance was assessed for each participant by seating them two metres away from the experimenter. Participants were asked to fixate on the nose of the experimenter, extend their arms and bring their hands together in front of their eyes, leaving a small gap through which the participant could see the experimenter’s face. Through this gap, the experimenter could see only one of the participant’s eyes: the visible eye was recorded as dominant.

Participants were sat in a chinrest 57 cm away from the display, with a headband placed around the top of their head. Prior to experimentation, participants underwent a 9-point calibration procedure.

**Figure 2:**
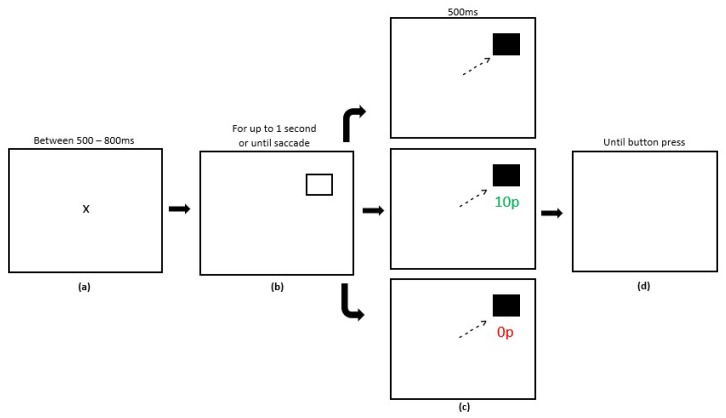
Sequence of events in the reward paradigm (adapted from Dunne et al., 2015 [31]; not to scale). (**a**) Participants were presented with a fixation cross for a variable time (500–800 ms). (**b**) The saccade goal was indicated by the appearance of a hollow square displayed for up to 1000 ms or until a saccade was made. (**c**) After a successful saccade or 1000 ms, participants received visual feedback in the colour change of the target stimulus. In the conditioning phase, when the target appeared at the rewarded location, successful saccades yielded a reward of 10 p on 60% of trials (Panel 2). No rewards were available when the target appeared at the unrewarded location, indicated by the presence of ‘0 p’. In the post-conditioning phase, no reward was available (Panel 3). Feedback was presented for 500 ms. (**d**) After a saccade towards the target, the trial ended and participants were required to press a button to start the next trial.

There were three experimental phases in the reward paradigm, Preconditioning (2 blocks, 120 trials), Conditioning (10 blocks, 600 trials) and Extinction (6 blocks, 360 trials). Each block contained 60 trials with the entire reward paradigm lasting 18 blocks. Participants were required to fixate centrally prior to the start of each trial. A variable fixation time period between 500–800 ms was programmed, after which a target stimulus square would appear in either the left or right hemifield. Participants had an upper limit of 1000 ms to make a saccade. A successful saccade to the target stimulus led to the stimulus changing colour from black to grey. After 500 ms, participants were presented with a blank screen and a button press was required to start the next trial. In the preconditioning phase, participants received no reward or reward feedback. In the conditioning phase, participants received rewards for successful saccades made towards one hemifield only. A variable-ratio reward schedule was employed whereby only 180 trials of the 300 trials to the rewarded hemifield were rewarded (60%). On rewarded trials and after a successful saccade, participants would receive feedback that they had accrued a monetary reward for the eye movement in the form of green text of ’10 p’ presented in Arial font. On unrewarded trials, participants saw red text of ‘0 p’. Trials in the post-conditioning phase of the experiment were identical to unrewarded trials in the conditioning phase where reward was removed entirely. Figure 2 displays the experimental array.

**Figure 3:**
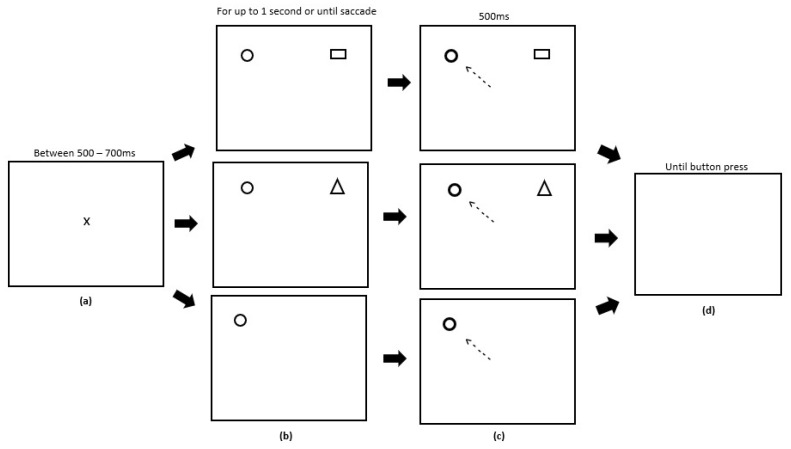
Sequence of events used in the remote distractor (RD) task (not to scale). (**a**) Participants were presented with a fixation cross of a variable time limit between 500–700 ms. (**b**) In a single block, participants could be presented with one of three trials: 1) a known distractor trial (Panel 1), where the distractor (square) used was the same stimuli associated with reward feedback in the reward paradigm; 2) a novel distractor trial (Panel 2), where the distractor (triangle) used was a novel stimulus; 3) a no distractor trial (Panel 3), where the target (circle) was presented on its own with no distractors. These were displayed for up to 1000 ms or until a saccade was made. (**c**) After a successful saccade to the target circle, this target circle would change colour (Panels 1, 2 and 3) and remain on screen for 500 ms. (**d**) Participants were presented with a blank screen, indicating that the trial had ended and they were required to press a button to start the next trial.

The RD task ran for six blocks directly after the conditioning and extinction phases of the reward paradigm. Each block contained 90 trials equally split between each condition type. Trials were also randomised. Participants were instructed to fixate on the central fixation cross prior to the start of each trial which appeared for a random period of time between 500 and 700 ms to avoid anticipatory eye movements. During a trial, participants had an upper limit of 1000 ms to make a saccade. After a successful saccade, the target stimuli would change colour from grey to white and would remain displayed for 500 ms, after which the trial ended. A button press was required to start the next trial. A single RD block consisted of three types of distractor trial: (1) a known distractor trial, consisting of a target circle in one hemifield and a distractor square (previously used as the target in the reward paradigm) in the opposite hemifield; (2) a novel distractor trial, consisting of a target circle in one hemifield and a novel stimulus (triangle) in the opposite hemifield; (3) a no distractor trial, where only a target circle appeared in one hemifield, with no other stimuli present. Figure 3 displays the experimental array.

#### 2.4.2. Experiment 2

**Figure 4:**
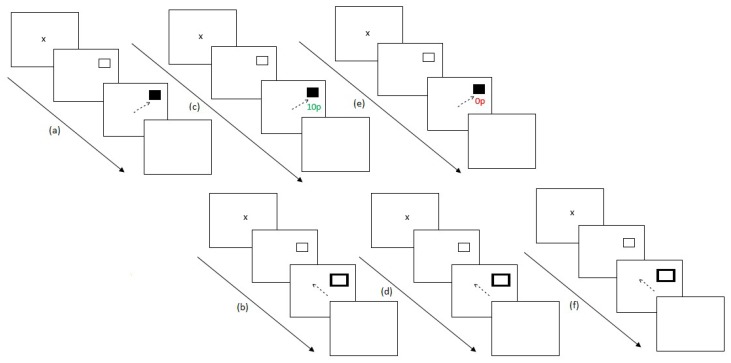
Experimental procedure for Experiment 2. (**a**) After assessing for eye dominance and a 9-point calibration procedure, participants completed two baseline blocks of the reward paradigm where no reward feedback was displayed. (**b**) Participants then completed six blocks of the antisaccade task, (**c**) followed by 10 conditioning blocks of the reward paradigm where only one hemifield was rewarded at a 60% schedule. (**d**) Participants then switched tasks again, completing six blocks of the remote distractor task. (**e**) A further six blocks of the reward paradigm were completed where no reward was given to participants for saccades. (**f**) Finally, participants completed six blocks of the remote distractor task and the experiment ended.

Experiment 2 ran similarly to Experiment 1 with the addition of a post-preconditioning phase of the secondary task to understand participant’s performance at baseline for the task. Experiment 2 lasted for 36 blocks. Firstly, participants completed the preconditioning phase of the reward paradigm (2 blocks). Participants then completed the post-preconditioning phase of the antisaccade task (6 blocks). This additional experimental block was added in order to understand participant’s baseline scores on the secondary unrewarded eye movement task. Participants then completed the conditioning phase of the reward paradigm (10 blocks) followed by the post-conditioning phase of the antisaccade task (6 blocks). Participants then completed the extinction phase of the reward paradigm (6 blocks) and finally the post-extinction phase of the antisaccade task (6 blocks). Figure 4 illustrates the experimental procedure.

**Figure 5:**
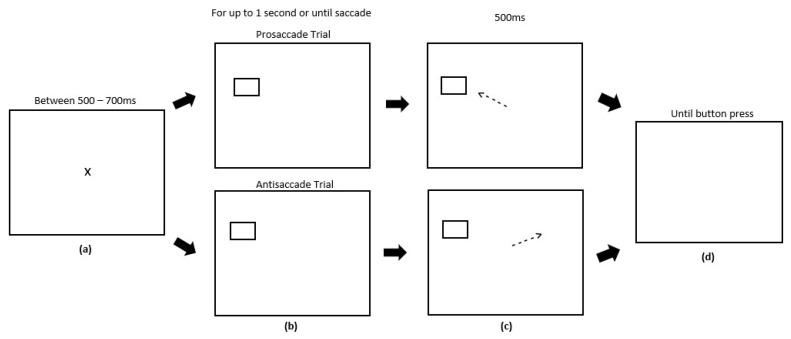
Sequence of events used in the antisaccade task (not to scale). (**a**) Participants were presented with a fixation cross for a variable time limit between 500–700 ms. (**b**) Participants were presented with a target square to either the left or right hemifield which displayed for 1000 ms or until a saccade was made (Panels 1 and 2). (**c**) On prosaccade trials, participants were required to saccade towards the target (Panel 1). On antisaccade trials, participants were required to saccade away from the target (Panel 2). After making a saccade, the target would embolden and be displayed for 500 ms. (**d**) Participants were presented with a blank screen after 500 ms. A button press was required to begin the next trial.

In Experiment 2, the reward paradigm was unchanged. The antisaccade task was run for 6 blocks and consisted of three experimental phases; (1) the Post-Preconditioning phase, which ran directly after the preconditioning phase of the reward paradigm; (2) the Post-Conditioning phase, which ran directly after the conditioning phase of the reward paradigm; (3) the Post-Extinction phase, which ran directly after the extinction phase of the reward paradigm. Figure 5 illustrates the experimental array. Each block contained 60 trials evenly split between randomised left antisaccade, right antisaccade, left prosaccade and right prosaccade trials. Participants were instructed to fixate on the central fixation cross prior to the start of each trial which displayed for a variable time limit between 500–700 ms. A blue fixation cross corresponded to a prosaccade trial, whereas a purple cross corresponded to an antisaccade trial. A target stimulus square would be presented to either the left or right hemifield for up to 1000 ms or until a saccade was made. After a successful saccade, the target stimuli would change colour from white to black and be presented for 500 ms, after which the trial ended and participants were presented with a blank screen. A button press was required to start the next trial.

### 2.5. Saccade Analysis

Mean SRT for each participant was calculated from each individual block of trials. Trials over 500 ms and saccadic errors made by participants were removed from the analysis; saccadic error is defined as trials where participant’s saccades left the fixation area but did not land at the target location.

#### 2.5.1. Experiment 1

In the 12,960 Reward Paradigm trials, 2.9% were categorised as saccadic errors. A further 9.7% of trials were above the threshold and also removed from the analysis. Of the 3240 RD task trials, 11.8% were categorised as saccadic errors and 4.6% of trials were above the threshold and so removed from the analysis.

#### 2.5.2. Experiment 2

Across 12,960 Reward Paradigm trials, 3.7% were categorised as saccadic errors. A further 6.8% of trials were above the threshold and also removed from the analysis. Of the 6480 prosaccade trials within this experimental phase, 9.6% were categorised as saccadic errors and 6.8% of trials were above the threshold. Of the 6480 antisaccade trials, 13.5% were identified as inaccurate and 9.3% were found to be above the threshold and so removed from the analysis.

## 3. Results

### 3.1. Latency

#### 3.1.1. Experiment 1

##### Reward Paradigm

The effect of rewards on saccade latency were assessed with a 3 (Experimental Phase: Preconditioning/Conditioning/Extinction) × 2 (Hemifield: Rewarded/Unrewarded) repeated-measures ANOVA on mean saccadic reaction times. This analysis revealed a main effect of Experimental Phase, (F(2,22) = 4.36, *p* = < 0.03, η^2^ = 0.284) such that saccades made during the conditioning phase (M = 191 ms, SD = 23.31) where rewards were present were significantly faster than saccades made during the extinction phase (M = 220 ms, SD = 27.80; *t*(11) = −2.93, *p* = < 0.017, *g* = 0.956) phase. No significant differences were found between the latency of saccades made during the preconditioning phase (M = 217 ms, SD = 35.28) compared to those made during the conditioning (M = 191 ms, SD = 23.31; *t*(11) = 1.84, *p* = 0.094, *g* = 0.870) or extinction phases (M = 220 ms, SD = 27.80; *t*(11) = −0.44, *p* = 0.667, *g* = 0.095). No effect of Hemifield was found (Rewarded: M = 205 ms, SD = 24.18; Unrewarded: M = 215 ms, SD = 19.07; F(1,11) = 3.49, *p* = 0.09, η^2^ = 0.241) but there was an interaction between Experimental Phase and Hemifield (F(2,22) = 5.23, *p* = 0.01, η^2^ = 0.322).

**Figure 6:**
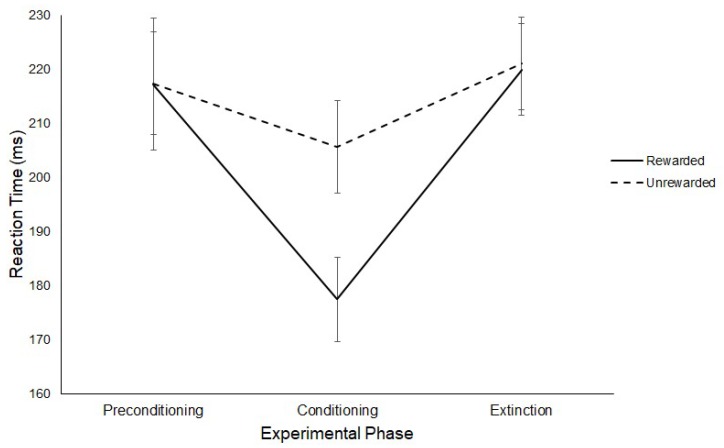
Latency of prosaccades to the rewarded and unrewarded hemifields across the preconditioning, conditioning and extinction phases of the reward paradigm in Experiment 1. Error bars show +/−1 SEM.

Three paired sample t-tests were conducted on the latency of saccades for the rewarded and unrewarded hemifields at each level of Experimental Phase. No significant differences were found between the latencies of saccades to the rewarded (M = 217 ms, SD = 42.27) or unrewarded hemifield (M = 217 ms, SD = 32.79) in the preconditioning phase (*t*(11) = −0.02, *p* = > 0.017, *g* = 0.005). A significant difference was found between the latencies of saccades to the rewarded (M = 177ms, SD = 27.06) and unrewarded (M = 206 ms, SD = 29.79) hemifields in the conditioning phase (*t*(11) = −2.99, *p* = < 0.017, *g* = 0.987), such that participants produced significantly faster SRTs towards the rewarded hemifield. No significant differences were found between the saccadic latencies in the rewarded (M = 220 ms, SD = 29.05) and unrewarded (M = 221 ms, SD = 29.55) hemifields for the extinction phase (*t*(11) = −0.20, *p* = > 0.017, *g* = 0.037). Figure 6 illustrates this result.

##### RD Task

To assess whether the effects of reward transferred to the RD task, a 3 (Distractor: Known/Novel/None) × 2 (Experimental Phase: Post-Conditioning/Post-Extinction) × 2 (Hemifield: Rewarded/Unrewarded) repeated-measures ANOVA on mean SRTs was conducted. This analysis revealed a main effect of Distractor (F (2,22) = 67.70, *p* = < 0.01, η^2^ = 0.860) such that trials in which there was no distractor (M = 201 ms, SD = 9.26) produced significantly faster saccades compared to known (M = 223 ms, SD = 9.49; *t*(11) = 8.40, *p* = < 0.017, *g* = 0.362) and novel (M = 222 ms, SD = 8.71; *t*(11) = 10.79, *p* = < 0.017, *g* = 0.451) distractor trials. No significant difference was found between the latency of saccades to known (M = 223 ms, SD = 9.49) and novel (M = 222 ms, SD = 8.71; *t*(11) = 0.49, *p* = 0.631, *g* = 0.110) distractor trials.

A main effect of Experimental Phase was also revealed (F(1,11) = 10.70, *p* = < 0.01, η^2^ = 0.493) such that saccades made in the post-extinction phase (M = 211 ms, SD = 8.47) were significantly faster than those in the post-conditioning phase (M = 220 ms, SD = 9.74). No other significant effects or interactions were found.

**Figure 7:**
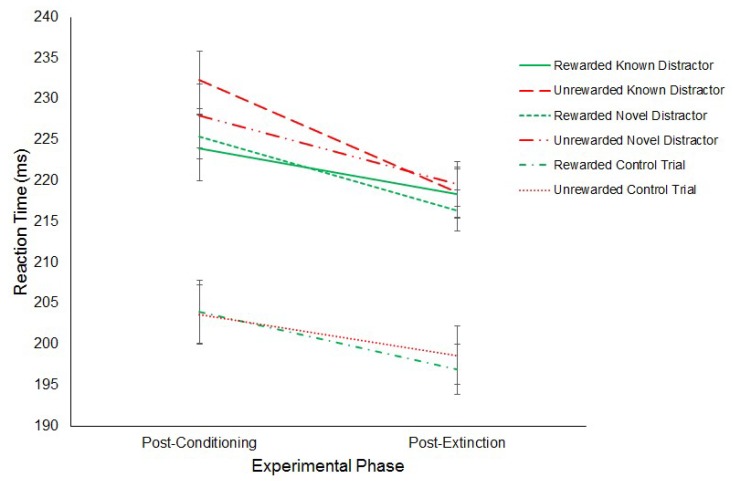
Latency of rewarded and unrewarded distractor trials across the post-conditioning and post-extinction phases of the remote distractor task in Experiment 1. Error bars show +/−1 SEM.

Interestingly, no effect of Hemifield was found (Rewarded: M = 214 ms, SD = 8.07; Unrewarded: M = 216 ms, SD = 8.92; F(1,11) = 2.68, *p* = 0.13, η^2^ = 0.196). Furthermore, no interaction effects were found (Distractor and Phase: F(2,22) = 1.46, *p* = 0.25, η^2^ = 0.118; Distractor and Hemifield: F(2,22) = 6.72, *p* = 0.52, η^2^ = 0.058; Phase and Hemifield: F(1,11) = 0.59, *p* = 0.46, η^2^ = 0.051; Distractor, Phase and Hemifield: F(2,22) = 3.38, *p* = 0.052, η^2^ = 0.235). Figure 7 displays this result.

In order to understand whether the findings were simply down to a wash-out effect whereby the time between trials post-reward were interfering with any post-reward saccade facilitation in the secondary task, an additional 3 (Distractor: Known/Novel/None) × 2 (Experimental block: 1–3/4–6) × 2 (Hemifield: Rewarded/Unrewarded) repeated-measures ANOVA on mean SRTs was conducted on binned latency data from blocks 1–3 and blocks 4–6 in the post-conditioning phase of the RD task. The analysis revealed a significant main effect of Distractor (Known: M = 259 ms, SD = 17.31; Novel: M = 260 ms, SD = 18.09; None: M = 215 ms, SD = 17.18; F(2,22) = 156.44, *p* = < 0.01, η^2^ = 0.934) such that no distractor trials were significantly faster than either distractor trials.

No significant main effect of Experimental Block (1–3: M = 246 ms, SD = 18.96; 4–6: M = 243 ms, SD = 16.02; F(1,11) = 1.36, *p* = 0.27, η^2^ = 0.110) or Hemifield (Rewarded: M = 242 ms, SD = 17.66; Unrewarded: M = 246 ms, SD = 16.61; F(1,11) = 3.44, *p* = 0.09, η^2^ = 0.238) was found. Furthermore, no significant interactions were found (Distractor and Experimental Block: F(2,22) = 3.59, *p* = 0.06, η^2^ = 0.246; Experimental Block and Hemifield: F(1,11) = 0.03, *p* = 0.86, η^2^ = 0.003; Distractor and Hemifield: F(2,22) = 0.40, *p* = 0.67, η^2^ = 0.035; Distractor, Experimental Block and Hemifield: F(2,22) = 0.60, *p* = 0.56, η^2^ = 0.051).

#### 3.1.2. Experiment 2

##### Reward Paradigm

The effect of rewards on saccade latency were assessed with a 3 (Experimental Phase: Preconditioning/Conditioning/Extinction) × 2 (Hemifield: Rewarded/Unrewarded) repeated-measures ANOVA on mean saccadic reaction times. Contrary to Experiment 1, no significant effect of Phase was revealed (Preconditioning: M = 245 ms, SD = 21.33; Conditioning: M = 238 ms, SD = 10.74; Extinction: M = 245 ms, SD = 15.05; F(2,22) = 2.26, *p* = 0.13, η^2^ = 0.170) This analysis revealed a main effect of Hemifield (F(1,11) = 5.61, *p* ≤ 0.04, η^2^ = 0.338), such that saccades made to the rewarded hemifield (M = 239 ms, SD = 11.37) were significantly faster than those made towards the unrewarded hemifield (M = 247 ms, SD = 19.12). Furthermore, an interaction between Phase and Hemifield was found (F(2,22) = 4.24, *p* ≤ 0.03, η^2^ = 0.278).

**Figure 8:**
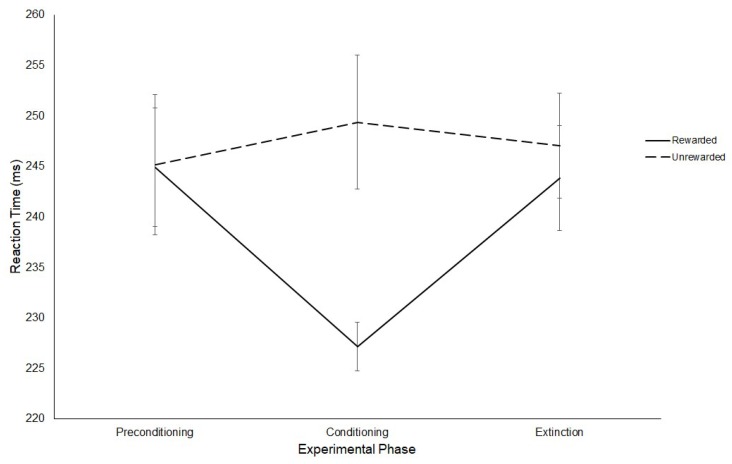
Latency of prosaccades to the rewarded and unrewarded hemifields across the preconditioning, conditioning and extinction phases of the reward paradigm in Experiment 2. Error bars show +/−1 SEM.

To explore this interaction, three paired sample t-tests were conducted on the latency of saccades for the rewarded and unrewarded hemifields at each level of Experimental Phase. These comparisons revealed no significant difference between the latencies of saccades to either hemifield in the preconditioning phase (Rewarded: M = 244 ms, SD = 20.25; Unrewarded: M = 245 ms, SD = 24.08; *t*(11) = −0.07, *p* = 0.95, *g* = 0.011). In contrast, a significant difference was found between the latencies of saccades to the rewarded (M = 227 ms, SD = 8.32) and unrewarded (M = 249 ms, SD = 22.96) hemifields in the conditioning phase (*t*(11) = −2.81, *p* ≤ 0.017, *g* = 0.342), such that participants produced significantly faster SRTs towards the rewarded hemifield. There were no significant differences between the saccadic latencies in the rewarded (M = 244 ms, SD = 17.97) and unrewarded (M = 247 ms, SD = 17.95) hemifields for the extinction phase (*t*(11) = −0.57, *p* = 0.58, *g* = 0.172). These results are displayed in Figure 8.

##### Antisaccade Task

A 3 (Experimental Phase: Post-Preconditioning/Post-Conditioning/Post-Extinction) × 2 (Saccade: Antisaccade/Prosaccade) × 2 (Hemifield: Rewarded/Unrewarded) repeated-measures ANOVA on SRTs revealed a main effect of Saccade (F(1,11) = 21.44, *p* ≤ 0.01, η^2^ = 0.661) with prosaccades (M = 240 ms, SD = 19.01) being significantly faster than antisaccades (M = 290 ms, SD = 33.36).

**Figure 9:**
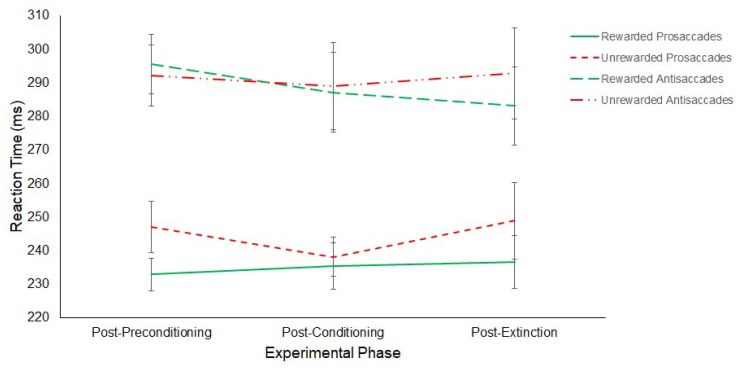
Latency of prosaccades and antisaccades to the rewarded and unrewarded hemifields across the post-preconditioning, post-conditioning and post-extinction phases of the reward paradigm in Experiment 2. Error bars show +/−1 SEM.

No main effect of Hemifield (Rewarded: M = 261 ms, SD = 19.01; Unrewarded: M = 268 ms, SD = 33.36; F(1,11) = 3.74, *p* = 0.08, η^2^ = 0.254) or Phase (Post-preconditioning: M = 266 ms, SD = 18.27; Post-conditioning: M = 263 ms, SD = 23.71; Post-extinction: M = 265 ms, SD = 30.74; F(2,22) = 0.18, *p* = 0.84, η^2^ = 0.016) were found. Furthermore, no interaction between Phase and Saccade (F(2,22) = 0.68, *p* = 0.52, η^2^ = 0.058), Saccade and Hemifield (F(1,11) = 1.37, *p* = 0.27, η^2^ = 0.110), Phase and Hemifield (F(2,22) = 1.57, *p* = 0.23, η^2^ = 0.125) or three-way interaction between Experimental Phase, Saccade Type and Hemifield (F(2,22) = 1.02, *p* = 0.38, η^2^ = 0.085) were revealed. The results are displayed in Figure 9.

Similarly to the RD task data, to understand whether the findings were due to a rapid wash-out effect, an additional 2 (Saccade: Prosaccade/Antisaccade) × 2 (Experimental block: 1–3/4–6) × 2 (Hemifield: Rewarded/Unrewarded) repeated-measures ANOVA on mean post-conditioning phase SRTs was conducted on binned latency data from blocks 1–3 and blocks 4–6 in the antisaccade task. The analysis revealed a significant main effect of Saccade (Prosaccade: M = 237 ms, SD = 19.43; Antisaccade: M = 288 ms, SD = 41.89; F(1,11) = 15.64, *p* = < 0.05, η^2^ = 0.587).

No main effect of Experimental Block (1–3: M = 261 ms, SD = 28.63; 4–6: M = 264 ms, SD = 21.43; F(1,11) = 0.36, *p* = 0.56, η^2^ = 0.032) or Hemifield (Rewarded: M = 261 ms, SD = 23.54; Unrewarded: M = 263 ms, SD = 25.30; F(1,11) = 0.42, *p* = 0.53, η^2^ = 0.037) were found. No interaction effects were found (Saccade and Experimental Block: F(1,11) = 0.05, *p* = 0.82, η^2^ = 0.005; Saccade and Hemifield: F(1,11) = 0.01, *p* = 0.94, η^2^ = 0.001; Experimental Block and Hemifield: F(1,11) = 0.22, *p* = 0.65, η^2^ = 0.020; Saccade, Experimental Block and Hemifield: F(1,11) = 0.17, *p* = 0.69, η^2^ = 0.016).

### 3.2. Saccadic Error

#### 3.2.1. Experiment 1

##### Reward Paradigm

A 3 (Experimental Phase: Preconditioning/Conditioning/Extinction) × 2 (Hemifield: Rewarded/Unrewarded) repeated-measures ANOVA on the proportion of saccadic errors revealed a main effect of Experimental Phase (F (2, 22) = 9.25, *p* = < 0.01, η^2^ = 0.457), such that a significantly smaller proportion of errors occurred during the preconditioning phase (M = 0.89, SD = 0.67) compared to the conditioning (M = 4.92, SD = 4.75; *t*(11) = −3.28, *p* = < 0.017, *g* = 1.147) and extinction (M = 2.53, SD = 2.08; *t*(11) = −3.17, *p* = < 0.017, *g* = 1.025) phases respectively. No effect of Hemifield (Rewarded: M = 4.50, SD = 3.85; Unrewarded: M = 3.83, SD = 3.44; F(1,11) = 0.92, *p* = 0.36, η^2^ = 0.077) or interaction (F(2,22) = 1.47, *p* = 0.25, η^2^ = 0.118) was found.

##### RD Task

Using the proportion of saccadic errors, a 2 (Experimental Phase: Post-Conditioning/Post-Extinction) × 3 (Distractor: Known/Novel/None) × 2 (Hemifield: Rewarded/Unrewarded) repeated-measures ANOVA revealed a main effect of Distractor (F(2,22) = 43.97, *p* ≤ 0.001, η^2^ = 0.800) such that trials in which there was no distractor (M = 0.34, SD = 0.99) produced significantly less oculomotor capture than known (M = 0.80, SD = 0.97; *t*(11) = 5.62, *p* ≤ 0.017, *g* = 0.453) or novel (M = 0.95, SD = 0.79; *t*(11) = 10.12, *p* = < 0.017, *g* = 0.657) distractor trials. No other significant effect or interactions were found (Phase: Post-conditioning: M = 0.63, SD = 1.12; Post-extinction: M = 0.76, SD = 1.57; F(1,11) = 2.69, *p* = 0.13, η^2^ = 0.196; Hemifield: Rewarded: M = 0.71, SD = 1.71; Unrewarded: M = 0.68, SD = 1.95; F(1,11) = 0.04, *p* = 0.85, η^2^ = 0.003; Phase and Distractor: F(2,22) = 0.82, *p* = 0.46, η^2^ = 0.069; Phase and Hemifield: F(1,11) = 1.14, *p* = 0.31, η^2^ = 0.094; Distractor and Hemifield: F(2,22) = 0.19, *p* = 0.83, η^2^ = 0.017; Phase, Distractor and Hemifield: F(2,22) = 0.18, *p* = 0.84, η^2^ = 0.016).

#### 3.2.2. Experiment 2

##### Reward Paradigm

A 3 (Experimental Phase: Preconditioning/Conditioning/Extinction) × 2 (Hemifield: Rewarded/Unrewarded) repeated-measures ANOVA on the proportion of saccadic errors revealed no main effect of Experimental Phase (Preconditioning: M = 1.49, SD = 1.29; Conditioning: M = 1.50, SD = 1.96; Extinction: M = 1.18, SD = 1.39; F(2,22) = 1.49, *p* = 0.25, η^2^ = 0.119), Hemifield (Rewarded: M = 1.34, SD = 1.95; Unrewarded: M = 1.44, SD = 2.32; F(1,11) = 0.37, *p* = 0.56, η^2^ = 0.032) or interaction between Phase and Hemifield (F(2,22) = 0.50, *p* = 0.61, η^2^ = 0.044).

##### Antisaccade Task

A 2 (Saccade Type: Prosaccade/Antisaccade) × 3 (Phase: Post-Preconditioning/Post-Conditioning/Post-Extinction) × 2 (Hemifield: Rewarded/Unrewarded) repeated-measures ANOVA was conducted on the proportion of errors within the antisaccade task. This analysis revealed a significant effect of Saccade Type (Prosaccade: M = 0.19, SD = 0.55; Antisaccade: M = 1.20, SD = 3.12; F(1,11) = 9.70, *p* = 0.01, η^2^ = 0.561) such that a significantly larger proportion of errors occurred in antisaccade trials than prosaccade trials. No effect of Phase (Post-preconditioning: M = 0.69, SD = 1.32; Post-conditioning: M = 0.72, SD = 1.31; Post-extinction: M = 0.67, SD = 1.28; F(2,22) = 0.17, *p* = 0.85, η^2^ = 0.015) or Hemifield (Rewarded: M = 0.75, SD = 1.76; Unrewarded: M = 0.82, SD = 1.96; F(1,11) = 0.54, *p* = 0.48, η^2^ = 0.022) was found. No interaction effects were found (Phase and Hemifield: F(2,22) = 1.01, *p* = 0.38, η^2^ = 0.028; Phase and Saccade: F(2,22) = 2.74, *p* = 0.76, η^2^ = 0.024; Hemfield and Saccade: F(1,11) = 3.24, *p* = 0.09, η^2^ = 0.228; Phase, Hemifield and Saccade: F(2,22) = 1.01, *p* = 0.79, η^2^ = 0.085.

## 4. Discussion

The aim of these experiments was to examine the transfer of eye movement facilitation between a training task rewarding saccade direction to a more complex, unrewarded eye movement task. A significant facilitation of saccade reaction times directed to a rewarded location was found in both Experiments 1 and 2, replicating Dunne et al. (2015) [31]. However, this facilitation of SRTs did not transfer to the RD or antisaccade tasks. In Experiment 1, slower SRTs were recorded after rewards were presented, suggesting a relative reward-saccade fatigue and extinction of any facilitation previously found. Additionally, participants produced significantly more errors in phases of the reward paradigm subsequent to the presentation of rewards. This provides potential evidence of a speed–accuracy trade-off in a task involving the potential to earn monetary rewards consistent with findings suggesting that rewards can modulate accuracy performance [19]. This is in contrast to previous findings suggesting that the effects of monetary rewards are able to transcend the speed–accuracy trade-off [35]. However, these results were not replicated in Experiment 2. No effect of rewards was found in the accuracy of eye movements in the RD task or antisaccade tasks.

These experiments demonstrate a significant facilitation of saccadic reaction time for eye movements directed to a rewarded location compared to an unrewarded location. This finding, observed in both experiments, is consistent with evidence of the effect of reward on the oculomotor system in saccade–direction paradigms [31,32] such that there is a relative facilitation when rewards are presented to rewarded locations. Interestingly, this hemifield-specific effect failed to transfer to more complex tasks in which saccades were made in the presence of a distractor, or subjects had to inhibit a saccadic response to the target. Further to this, although there was a facilitation of saccades to rewarded locations when rewards were present, there was no consistent impact of incentives on saccade accuracy in either the reward paradigm, antisaccade or remote distractor tasks.

These data suggest that the effects of operant conditioning in the oculomotor system are task specific and as such do not transfer between tasks. This is consistent with the lack of hemifield-specific or, even generally, faster eye movements in the RD or antisaccade phase directly after the conditioning phase of the reward paradigm. Direct comparisons can be made between the reward paradigm trials, the no distractor trials in the RD task and the prosaccade trials in the antisaccade task. These trial types are very similar except for the shape of the target stimulus and the reward feedback available in the reward paradigm. No hemifield-specific effects of reward were found in the no distractor trials of the RD task, or prosaccade trials in the antisaccade task. It seems that the facilitation recorded in the conditioning phase of the reward paradigm is sensitive to a multitude of factors including alterations in task demands, changing stimuli and different trial types. Therefore, it is possible that the sensitivity of the facilitation effect of reward is susceptible to changes in the context that participants are rewarded in, failing to replicate when the context in which facilitation occurs is altered [36]. This suggests that the effects seen in the reward paradigm are task specific, consistent with our previous finding [31].

An additional explanation to consider is simply that the experimental protocol outlined in the present set of experiments led to a rapid wash-out effect of any saccade facilitation found in the reward paradigm. However, our previous research using a similar paradigm demonstrated that the facilitative effects of reward are present for approximately 180 trials [31]. As such, we would expect the effects of reward to be present for at least three blocks in the secondary tasks employed in Experiments 1 and 2. To address this issue, an additional analysis was conducted on mean SRT data for each participant from the first three blocks compared to the last three blocks of the task directly after the rewarded condition in both experiments. In the antisaccade task, there was no evidence that SRTs were systematically faster to the rewarded hemifield in the first three blocks than the second three blocks of the transfer task. The data from the RDE task were less clear. Although there were no statistically significant effects, the pattern of data suggests that the bias towards the rewarded hemifield may not have been entirely extinguished in the RDE task. However, the important point is that this bias did not interact with the RDE effect, consistent with our conclusion that the effects of reward are task specific. There was also a non-significant trend towards an interaction between Distractor Type and Experimental Block, perhaps hinting that the RDE effect differed in the early and late phases of the transfer task. However, there was no interaction with hemifield, suggesting that this marginal effect was independent of any effect related to reward.

The transient nature of the facilitation of saccades through operant conditioning contrasts starkly with the effects of rewarding objects on visuospatial attention, which have been reported to last for several days [22]. Previous studies had suggested that a stimulus or stimulus features associated with rewards are granted attentional priority. As such, they become more salient [14,21,22,23,37,38] triggering spatial attention towards it [23,39,40] or capturing attention non-spatially when attention already lingers at the spatial location of the stimulus [41]. In our study, this was not the case. The key methodological difference between these paradigms and the experiments presented in this paper is the difference between rewarding stimulus features and rewarding spatial locations. As such it may be the case that rewarding spatial locations produces a short-term, strategic bias toward the rewarded location rather than a sustained attentional prioritization of that location.

These results are consistent with the idea that the oculomotor system is highly adaptable, which allows it to rapidly learn which locations are most likely to yield reward but also extinguish this learning when the context changes. This idea is consistent with the evidence that reward signals do not influence the oculomotor preparation until relatively late in the process of saccade planning [42]. When taken together with the current findings, these data suggest that persistent biases in visual exploration triggered by rewarding particular stimulus features arise from changes in attentional prioritization of objects (termed ‘reward-dependent attentional learning’ by Chelazzi, Perlato, Santandrea, and Libera, 2013)) [24], not persistent changes in the oculomotor system.

Previous experimentation with rewards has focused on the potential uses of money as a viable rehabilitator in visual field deficits. Spatial neglect is an extremely common disorder of attention post-stroke. One potential avenue for intervention in neglect is the use of monetary rewards in order to negate the visual biases associated with this disorder. Malhotra, Soto, Li, and Russell (2012) have shown that omissions in a cancellation task were reduced for both left and right targets when patients searched for pictures of coins and were promised monetary rewards for every target found, relative to a no reward condition [26]. Furthermore, Lucas et al., (2013) investigated the specific effects of reward on spatial attention using a novel gambling task in a population of neuro-typical and neglect patients [43]. In a neuro-typical cohort, when rewards were available to both hemifields, no change in oculomotor behaviour was recorded. However, presenting high value rewards to one hemifield resulted in a progressive shift of target choices to that hemifield, correlating with the data presented in Experiments 1 and 2. In the patient sample, target choices gradually shifted to the impaired visual field, where the highest rewards were available. Although these findings are promising, the findings of the present study oppose these previous studies, suggesting the possibility that rewards may be used to help patients with brain injuries compensate for neuropsychological problems with attention and memory. The lack of transfer of facilitation from the trained oculomotor task to untrained cognitive tasks in neurotypical participants suggests that any interventions incorporating reward training of specific spatial locations may be of limited benefit for patient populations.

In summary, when reward feedback was available, participants were significantly faster at making saccades to rewarded locations, consistent with previous research [31]. However, this effect failed to transfer to more complex oculomotor behaviours. The data suggest that operant conditioning of eye movements to spatial locations produces rapid but highly task-specific learning, unlike operant conditioning of non-spatial features such as colour and shape. Based on these findings, rewarding eye movements to specific spatial locations is unlikely to induce long-term, systemic changes to the human action system.
